# Comparative impact of insect growth regulators on mortality and development of *Amrasca biguttula* (Hemiptera: Cicadellidae)

**DOI:** 10.1371/journal.pone.0350736

**Published:** 2026-06-05

**Authors:** Sabrine Attia, Shimat V. Joseph

**Affiliations:** 1 Department of Entomology, University of Georgia, Griffin, Georgia, United States of America; 2 Laboratory of Bioaggressors and Integrated Pest Management in Agriculture (LR14AGR02), National Agronomic Institute of Tunisia (INAT), University of Carthage, Tunis, Tunisia; University of Carthage, TUNISIA

## Abstract

The two-spot cotton leafhopper, *Amrasca biguttula* (Ishida) (Hemiptera: Cicadellidae), recently detected in the United States, represents an emerging threat to cotton, vegetable, and ornamental crops. Insect growth regulators (IGRs) are considered reduced-risk insecticides. Despite their availability to growers and effectiveness on several piercing and sucking insects, the lethal effects of IGRs on the development of *A. biguttula* remain poorly understood. Thus, the objective of this study was to determine the effects of common IGRs on various stages of *A. biguttula*. We evaluated four IGRs: pyriproxyfen, novaluron, azadirachtin, and buprofezin applied at field-recommended rates, alone or combined with nonionic and organosilicone adjuvants, on survival, molting disruption (exuviae production), and longevity of early (1^st^-2^nd^), intermediate (3^rd^-4^th^), and late (5^th^) nymphal instars, as well as adults using leaf dip and adaxial leaf smear bioassays. All IGRs induced significant, stage-dependent lethal effects. Mortality of 1^st^–2^nd^ instars reached over 90% with buprofezin and novaluron, and molting inhibition reached up to 55%, indicating strong effects of the tested insecticides. The chitin biosynthesis inhibitors buprofezin and novaluron caused rapid mortality, strong molting inhibition, and reduced longevity, particularly in early and intermediate instars. Pyriproxyfen and azadirachtin elicited weaker, delayed responses, with limited effects on late instars and adults. Although adding adjuvants slightly enhanced efficacy, their overall impact was marginal. These findings demonstrate that IGRs can profoundly disrupt *A. biguttula* population development through interference with insect growth and metamorphosis, supporting their use as selective and sustainable tools in integrated pest management programs targeting this invasive leafhopper.

## Introduction

Biological invasions by alien insect species represent a major and rapidly intensifying driver of global agroecosystem disruption, threatening crop productivity, biodiversity, and ecosystem stability worldwide [1–4]. Invasive insects often display exceptional ecological plasticity, allowing them to establish, spread, and persist in novel environments despite strong biotic and abiotic filters [5]. Such success is commonly linked to life-history traits, including high reproductive output, short generation time, broad host range, and tolerance to environmental variability, traits that collectively promote population outbreaks and severe economic impacts in invaded regions [4,6,7].

Among invasive hemipteran pests, leafhoppers (Hemiptera: Cicadellidae), have emerged as particularly problematic due to their high mobility, multivoltine life cycles, and piercing–sucking feeding behavior [8]. Both nymphs and adults damage plants by extracting sap and injecting phytotoxic saliva, resulting in chlorosis, necrosis, leaf curling, and the characteristic “hopper burn” symptoms that reduce photosynthetic efficiency and plant vigor [9–12]. In invaded agroecosystems, leafhopper outbreaks are often favored by warm climatic conditions and intensive crop management, suggesting that ongoing climate change and agricultural intensification may further enhance their invasive potential [13].

The two-spot cotton leafhopper, *Amrasca biguttula* (Ishida) (Hemiptera: Cicadellidae), is an economically important invasive leafhopper species of increasing concern. Native to the Indian subcontinent, with a historical distribution extending from Iran to Japan and Southeast Asia, recent phylogeographic analyses have clarified its invasion history, revealing a single-haplotype incursion into the United States beyond its putative native range [14]. It has long been recognized as a destructive pest of cotton (*Gossypium hirsutum* L.), okra (*Abelmoschus esculentus* (L.) Moench), eggplant (*Solanum melongena* L.), and numerous other cultivated and ornamental plant species [12]. Under severe infestation conditions, yield losses can exceed 40%, primarily due to direct feeding damage [15]. More recently, the detection and rapid spread of *A. biguttula* in the United States indicate a significant expansion beyond its previously known distribution, raising serious concerns for both field crops and high-value ornamental production systems.

The invasion success of *A. biguttula* is tightly linked to its life-history traits and reproductive biology [8]. Females oviposit eggs within leaf tissues, protecting developing stages from abiotic stressors, natural enemies, and insecticide exposure, thereby allowing early population establishment to occur undetected and increasing the likelihood of successful colonization in new environments. Rapid embryonic and nymphal development enables multiple overlapping generations within a single growing season, resulting in exponential population growth [16]. Moreover, the broad host range of *A. biguttula* supports its persistence across diverse agroecosystems and promotes dispersal through the movement of infested plant material, particularly in ornamental systems and nursery trade networks [12].

In invaded regions, control of leafhopper populations has primarily relied on repeated applications of broad-spectrum insecticides, such as bifenthrin, thiamethoxam, afidopyropen, flupyradifurone, and sulfoxaflor, to rapidly suppress pests [17]. Insecticide-based strategies can effectively reduce pest densities in the short term, but they may also impose strong selective pressures that accelerate the development of insecticide resistance and disrupt natural enemy communities in arthropods [7,18]. In agroecosystems, pesticide exposure often occurs under variable conditions, which can influence treatment efficacy and pest population dynamics. [7]. Ecotoxicological studies have traditionally evaluated insecticide efficacy primarily by measuring acute mortality in target pests; however, conventional insecticides can have multiple adverse impacts, including toxicity to non-target organisms, environmental contamination, resistance development, and disruption of ecosystem services [19]. Despite being relatively inexpensive and accessible to growers, these limitations highlight the urgent need for safer and more sustainable pest management strategies.

In this context, insect growth regulators (IGRs) represent a promising class of insecticides. By interfering with molting, metamorphosis, and reproductive processes, IGRs can exert strong population-level effects while reducing negative impacts on non-target organisms and beneficial arthropods [20]. Their mode of action makes them particularly suitable for pests with rapid developmental cycles and concealed immature stages, such as *A. biguttula*, whose eggs are partially protected within leaf tissues. However, despite these advantages, the ecotoxicological effects of IGRs on invasive leafhopper species, particularly at lethal doses, remain largely unexplored.

To date, no study has comprehensively evaluated the lethal effects of insect growth regulators (IGRs) on *A. biguttula*. Given the pest’s recent establishment, high reproductive potential, and increasing reliance on chemical control, such information is urgently needed to support evidence-based management decisions. Therefore, the objective of this study was to assess the lethal effects of selected IGRs on *A. biguttula*, with particular emphasis on survival, development, and molting. By focusing on these key life-history parameters, this work aims to contribute to the development of sustainable and effective integrated pest management (IPM) strategies against this emerging invasive leafhopper.

## Materials and methods

### Plant and insects

Laboratory colonies of *A. biguttula* were initially established from adults collected in an okra field in Tifton, Georgia, USA (31°29′12″N, 83°31′08″W) on 30 September 2025. The colony was subsequently maintained on hibiscus (*Hibiscus rosa-sinensis* L.; Malvaceae) plants grown in 3.7 L plastic containers under greenhouse conditions at the University of Georgia Griffin Campus, Griffin, Georgia, USA. Insects used in the experiments were obtained from this colony after being reared for three consecutive generations under controlled conditions. Only young, intermediate-sized leaves from the upper shoots were used to ensure consistent nutrition. Plants were purchased from a local nursery and had not been exposed to pesticides before purchase. Heavily infested plants were replaced monthly to maintain colony health.

In the greenhouse, plants infested with *A. biguttula* were kept in insect-rearing cages (60 [W] × 60 [D] × 91 [H] cm, Butterfly Habitat XL, RestCloud, Hangzhou, China) wereused to enclose the containers. Colonies were maintained at 50% relative humidity (RH) and 26 ± 2 °C, with a 12L:12D h photoperiod.

### Experimental design and environmental conditions

All bioassays were conducted in environmentally controlled chambers (Percival Scientific, Perry, IA, USA; chamber number A06840730) maintained at 28 °C and 50% RH under a 16L:8D h photoperiod. Hibiscus plants used in the bioassays were maintained without fertilizer and were irrigated manually once daily.

Two bioassay protocols were conducted to evaluate the efficacy of selected IGRs. The leaf dip assay without adjuvants exposed multiple insect stages directly to treated leaves, whereas the leaf smear assay with adjuvants targeted nymphal stages under conditions that simulated typical field applications. During insecticide spraying, residues are primarily deposited on the adaxial surface of the leaves. Leaves were air-dried (approximately 10 min) until no visible moisture remained before insects were introduced. These protocols are presented in separate subsections to clearly distinguish their methodology and objectives.

For various experiments, *A. biguttula* individuals were assigned to one of the four groups. The groups were 1) 1^st^-2^nd^, 2) 3^rd^-4^th^, 3) 5^th^ instars) and 4) adults. The groups were created based on morphological similarity and size, thereby minimizing stage-specific errors. Insects were randomly assigned to treatments and replicates, and Petri dishes were arranged randomly within the environmental chamber to minimize positional bias. Leaves were selected based on uniform age, size, and position (upper canopy) to ensure consistency across treatments. Adult *A. biguttula* used in the experiments were not sexed, and exact adult age was not standardized. To minimize variability, only healthy and active adults of similar size and appearance from the same laboratory generation were randomly selected and maintained under identical environmental conditions before bioassays.

### Insecticides

All experiments evaluated mortality at the maximum labeled rates of the insect growth regulators (IGRs) used in this study: azadirachtin (8.4 g ha ⁻ ¹), pyriproxyfen (90.3 g ha ⁻ ¹), novaluron (58.1 g ha ⁻ ¹), and buprofezin (235 g ha ⁻ ¹) (Table 1). Spray volume was 935.4 L ha ⁻ ¹, resulting in final concentrations of 238.7, 153.6, 22.2, and 620.4 ppm, respectively (Table 1). Insecticide solutions were prepared by diluting commercial formulations in distilled water to obtain the desired concentrations based on the maximum labeled rates. Solutions were freshly prepared before each experiment and thoroughly mixed to ensure homogeneity, following standard procedures similar to those recommended by the Insecticide Resistance Action Committee (IRAC).

**Table 1:** Details of the insect growth regulator applied to *A. biguttula* in trials 1 and 2.

Brand name	Active ingredient (AI)	a.i^a^ (%)	IRAC^b^ group	Manufacturer	Target pests	Application rate (U.S)	Rate^c^ (g a.i/ ha ⁻ ¹)	Final concentration (ppm)
Azatin® O	Azadirachtin	1.8% (18 g L ⁻ ¹)	UN (Botanical IGR)	Certis/ OHP	Aphids, thrips, leafminers, whiteflies, fungus gnats	16 fl oz/ 100 gal	8.4	22.2
Fulcrum®	Pyriproxyfen	11.23% (112.3 g L ⁻ ¹)	7C (Juvenile hormone analog)	OHP Inc.	Whiteflies, aphids, scales, fungus gnats, shore flies	12 fl oz/ 100 gal	90.3	238.7
Pedestal®	Novaluron	10.0% (100 g L ⁻ ¹)	15 (Chitin biosynthesis inhibitor)	ADAMA/ OHP	Caterpillars, thrips, whiteflies, stink bugs	8 fl oz/ 100 gal	58.1	153.6
Talus® 70 DF	Buprofezin	70% (700 g kg ⁻ ¹)	16 (Chitin biosynthesis inhibitor)	SePRO Corp.	Whiteflies, mealybugs, scales, planthoppers	12 oz/ 100 gal	235	620.4

^**a**^Active ingredient; ^b^Insecticide resistance Action Committee; ^c^Insect growth regulator solution was prepared in 378.54 L per ha of water.

### Bioassays

#### Leaf dip assay without adjuvant.

Two experiments were conducted using this protocol. The treatments for both experiments were 1) azadirachtin, 2) pyriproxyfen, 3) novaluron, 4) buprofezin, and 5) non-treated control. Leaves were dipped in IGR solutions for 5 s, air-dried, and placed in Petri dishes with the abaxial surface down. Petri dishes were sealed with Parafilm® to maintain humidity.

Experiment 1. Ten individuals of the same developmental stage were placed in each 90 mm × 15 mm Petri dish, with ten replicates per treatment for 1) 1^st^ −2^nd^, 2) 3^rd^-4^th^, and 3) 5^th^ instars. For adults, there were only five replications. Each Petri dish was considered an independent experimental unit. Leaf treatment and data recording were identical to Experiment 1. Mortality and exuviae were recorded daily for 7 d and 6 d in trials 1 and 2, respectively. Trials 1 and 2 were conducted from 21 to 28 October and from 21 to 28 November 2025.

Experiment 2. Four individuals, one per developmental stage, were placed in each 90 mm × 15 mm Petri dish. Each treatment was replicated five times. Longevity was recorded daily for 7 d. Longevity was defined as the number of days individuals survived from the start of the bioassay until death. Dead insects were confirmed by gentle probing. Trials 1 and 2 were initiated on 21 October and 23 October 2025.

#### Leaf smear assay with adjuvants.

The bioassay procedure was similar to that described in Experiments 1 and 2, with minor modifications. Only the two most effective IGRs, buprofezin and novaluron, identified in Experiments 1 and 2 were used for this assay. A follow-up bioassay was conducted to examine the translaminar activity of selected IGRs, applied alone or with adjuvants, against 1^st^–2^nd^ instars. This approach reflects field conditions, where nymphs typically reside on the abaxial leaf surface while insecticides are applied to the adaxial surface.

Two adjuvants were used: (1) a nonionic surfactant, (99% methyl esters of C16–C18 fatty acids, polyalkyleneoxide-modified polydimethylsiloxane, and alkylphenol ethoxylate (Dyne-Amic® Helena Agrichemicals, Collierville, TN, USA), and (2) a nonionic organosilicone surfactant, polyether-modified trisiloxane (Capsil® Aquatrols, Paulsboro, NJ, USA). Dyne-Amic® and Capsil® were added to the IGR treatments at concentrations of 0.1% and 0.25% (v/v), respectively. The spray volume used in the second trial was 935.4 L ha ⁻ ¹, consistent with standard nursery production practices.

The treatments were: (1) water control, (2) novaluron, (3) novaluron + Dyne-Amic®, (4) novaluron + Capsil®, (5) buprofezin, (6) buprofezin + Dyne-Amic®, (7) buprofezin + Capsil®, (8) Dyne-Amic®, and (9) Capsil®. Ten individuals per treatment per replicate were used, with ten replicates. Each container was considered an independent experimental unit. The assay consisted of a hibiscus leaf, with the leaf petiole dipped into a pool of water in a 120 mL plastic cup. To keep the leaf upright, the petiole was inserted through a 0.5-cm-diameter hole drilled in the plastic lid. The entire assay (leaf + cup) was placed inside the 2 L clear plastic container. The leaf petiole was dipped in water throughout the experiment to prevent the leaf from desiccating quickly. Mortality and molting were recorded daily for 8 days. Trials 1 and 2 were conducted from 21 to 28 October and from 21 to 28 November 2025.

### Statistical analyses

The data were analyzed using Statistical Analysis Software [21]. To assess the effects of IGRs, survival and exuviae count data were analyzed using a generalized linear mixed model (GLMM) with a Poisson error distribution and a log link function. Analyses were conducted separately for each stage for survival and exuviae count data. Treatment, time, and their interaction were included as fixed effects, while replication (rep) was modeled as a random effect to account for correlation among repeated observations. Model parameters were estimated using the Laplace approximation to the likelihood.

To evaluate the adequacy of the Poisson assumption, overdispersion was assessed using Pearson residuals. Pearson residuals were extracted from the fitted model, squared, and summed within each stage to obtain a Pearson chi-square statistic. This value was divided by the corresponding number of observations to compute a dispersion estimate. Dispersion estimates close to 1 were interpreted as indicating no substantial overdispersion.

To determine the effects of IGRs on *A. biguttula* longevity in the leaf dip assay, data were analyzed by 1^st^ −2^nd^, 3^rd^-4^th^, and 5^th^ instars using a generalized linear model in PROC GLIMMIX in SAS. Analysis was conducted separately for each trial. Analyses assumed a Poisson distribution, employing the Laplace method and a log link function. IGR treatment was treated as a fixed effect, whereas replication was treated as a random effect in the models.

To assess the effects of IGRs with and without adjuvants, time of exposure, and their interaction on *A. biguttula* survival and exuviae in the leaf smear assay, 1^st^-2^nd^ instar data were analyzed using a generalized linear model in PROC GLIMMIX in SAS. Analysis was conducted separately for each trial. Analyses assumed a Poisson distribution, employing the Laplace method and a log link function. Independent variables such as IGR and observation date were treated as fixed effects, whereas replication was treated as a random effect.

For all analyses, a value of 1 was added to each data point, including surviving *A. biguttula* individuals, exuviae, and longevity data, to address zero inflation. Means and standard errors for survival, exuviae, and longevity data were calculated using PROC MEANS, and means were separated using the Tukey-Kramer test (α = 0.05).

## Results

### IGR leaf dip assay without adjuvant and on varied *A. biguttula* stages

#### Survival.

**Fig 1:**
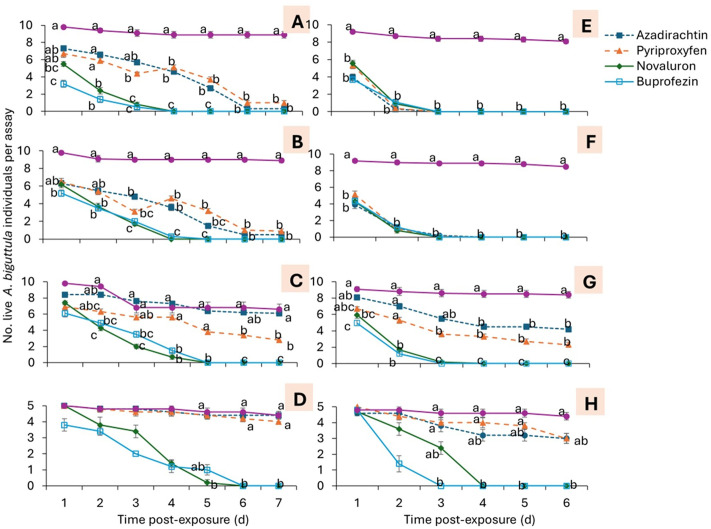
Mean (± SEM) number of *A. biguttula* survived by (A) 1^st^-2^nd^ instar, (B) 3^rd^-4^th^ instars, (C) 5^th^ instar, (D) adults for the trial 1, as well as (E) 1^st^-2^nd^ instar, (F) 3^rd^-4^th^ instars, (G) 5^th^ instar, (H) adults for the trial 2 in the leaf dip assay. Data points within each figure that share the same letters at the same post-exposure observation time for the treatments are not significantly different at α = 0.05 (Tukey-Kramer test). Where no differences were observed among treatments, no letters are given.

The effects of IGRs, time of exposure, and their interaction on *A. biguttula* were significant for the survival of 1^st^-2^nd^, 3^rd^-4^th^, 5^th^ instars, and adults in trials 1 and 2 (Table 2). In trial 1, the number of surviving 1^st^-2^nd^ instars was significantly lower in the buprofezin treatment than in the nontreated control, azadirachtin, and pyriproxyfen treatments at 1 d post-exposure (DPE) (Fig 1A). At 2 DPE, significantly fewer 1^st^-2^nd^ instars were observed in the buprofezin and novaluron treatments than in the nontreated control, azadirachtin, and pyriproxyfen treatments. At 4–5 DPE, the number of 1^st^-2^nd^ instars was significantly lower in the buprofezin and novaluron treatments than in the azadirachtin and pyriproxyfen treatments, followed by the nontreated control treatment (Fig 1A). At 6–7 DPE, all IGRs had significantly fewer 1^st^-2^nd^ instars than the nontreated control (Fig 1A). For 3^rd^-4^th^ instars, the number of surviving individuals was significantly lower in the buprofezin treatment than in the nontreated control, azadirachtin, and pyriproxyfen treatments at 1 DPE (Fig 1B). At 2 DPE, the number of 3^rd^-4^th^ instars was significantly lower in the buprofezin and novaluron treatments than in the nontreated control treatment (Fig 1B). At 4 DPE, the number of 3^rd^-4^th^ instars was significantly lower in the buprofezin and novaluron treatments than in the azadirachtin and pyriproxyfen treatments, followed by the nontreated control treatment (Fig 1B). At 6–7 DPE, all IGRs had significantly fewer 3^rd^-4^th^ instars than the nontreated control (Fig 1B). For 5^th^ instars, the number of surviving individuals was significantly lower in the novaluron treatment than in the nontreated control treatments at 2 DPE (Fig 1C). At 3–5 DPE, the number of 5^th^ instars was significantly lower in the buprofezin and novaluron treatments than in the nontreated control treatment (Fig 1C). At 6 and 7 DPE, the number of 5^th^ instars was significantly lower in the buprofezin and novaluron treatments than in the pyriproxyfen treatment, followed by the azadirachtin and nontreated control treatments (Fig 1C). At 5 DPE, significantly fewer adults were observed in the novaluron-treated group than in the nontreated control group (Fig 1D). At 6 and 7 DPE, the number of 5^th^ instars was significantly lower in the buprofezin and novaluron treatments than in the azadirachtin, pyriproxyfen, and nontreated control treatments (Fig 1D). There was no significant difference between azadirachtin, pyriproxyfen, and nontreated control treatments at 6 and 7 DPE.

**Table 2:** Statistical analysis of *A. biguttula* survival and exuviae generated after exposure to various IGRs in a leaf dip assay.

Treatment	Trial 1			Trial 2		
	*F*	df	*P*	*F*	df	*P*
Stages						
*1*^*st*^ *−2*^*nd*^ *instars*						
Insecticide	139.3	4,351	<0.001	170.4	4,356	<0.001
Time	75.9	7,351	<0.001	121.3	6,356	<0.001
Insecticide × Time	10.2	28,351	<0.001	12.9	24,356	<0.001
*3*^*rd*^ *−4*^*th*^ *instars*						
Insecticide	130.2	4,351	<0.001	176.5	4,356	<0.001
Time	77.2	7,351	<0.001	117.1	6,356	<0.001
Insecticide × Time	9.2	28,351	<0.001	12.7	24,356	<0.001
*5*^*th*^ *instar*						
Insecticide	81.3	4,351	<0.001	104.2	4,356	<0.001
Time	56.4	7,351	<0.001	73.9	6,356	<0.001
Insecticide × Time	6.7	28,351	<0.001	9.4	24,356	<0.001
*Adults*						
Insecticide	20.4	4,156	<0.001	19.9	4,161	<0.001
Time	8.0	7,156	<0.001	13.7	6,161	<0.001
Insecticide × Time	1.9	28,156	0.008	2.6	24,161	<0.001
Exuviae						
*1*^*st*^ *−2*^*nd*^ *instars*						
Insecticide	38.5	4,351	<0.001	55.1	4,356	<0.001
Time	9.2	7,351	<0.001	9.4	6,356	<0.001
Insecticide × Time	3.0	28,351	<0.001	3.2	24,356	<0.001
*3*^*rd*^ *−4*^*th*^ *instars*						
Insecticide	23.9	4,351	<0.001	43.9	4,356	<0.001
Time	9.0	7,351	<0.001	9.4	6,356	<0.001
Insecticide × Time	2.6	28,351	<0.001	3.2	24,356	<0.001
*5*^*th*^ *instar*						
Insecticide	6.3	4,351	<0.001	36.1	4,356	<0.001
Time	25.0	7,351	<0.001	18.5	6,356	<0.001
Insecticide × Time	2.1	28,351	0.001	3.5	24,356	<0.001

In trial 2, the numbers of surviving 1^st^-2^nd^ (Fig 1E) and 3^rd^-4^th^ instars (Fig 1F) were significantly lower across all IGR treatments than in the nontreated control at 1–6 DPE. The number of 5^th^ instars was significantly lower in the buprofezin treatment than in the nontreated control and azadirachtin treatments at 1 DPE (Fig 1G). At 2 DPE, the number of 5th instars was significantly lower in the buprofezin and novaluron treatments than in the azadirachtin, pyriproxyfen, and nontreated control treatments (Fig 1G). At 3 DPE, significantly fewer 5^th^ instars were observed in the buprofezin and novaluron treatments than in the pyriproxyfen treatment, which was followed by the nontreated control (Fig 1G). On the remaining observation dates, the number of 5th instars was significantly lower in the buprofezin and novaluron treatments than in the azadirachtin and pyriproxyfen treatments, with the nontreated control next (Fig 1G). For adults, significantly fewer individuals were observed in the buprofezin treatment than in the nontreated control, azadirachtin, and pyriproxyfen treatments at 3–5 DPE (Fig 1F). There was no significant difference between treatments at 1 and 2 DPE.

#### Exuviae.

**Fig 2:**
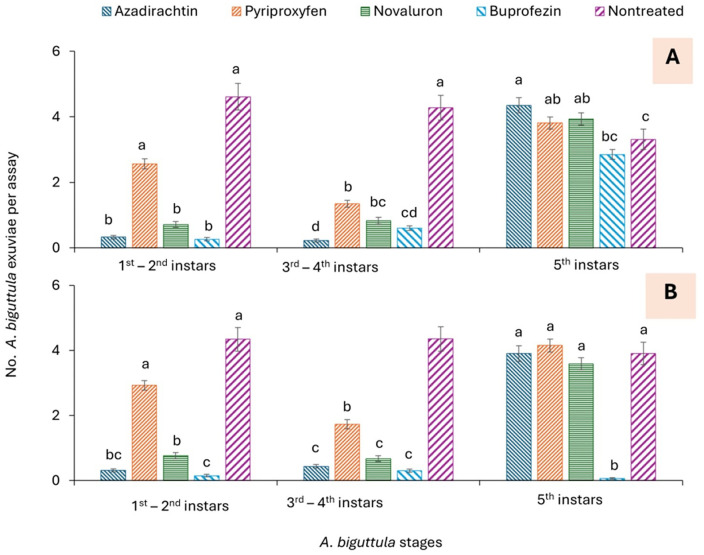
Mean (± SEM) number of *A. biguttula* exuviae for (A) trial 1 and (B) trial 2, by 1^st^-2^nd^ instar, 3^rd^-4^th^ instars, and 5^th^ instars in the leaf dip assay. Bars within each figure that share the same letters within each *A. biguttula* stage for the treatments are not significantly different at α = 0.05 (Tukey-Kramer test).

The effects of IGRs, time of exposure, and their interaction on *A. biguttula* were significant for exuviae of 1^st^-2^nd^, 3^rd^-4^th^, and 5^th^ instars in trials 1 and 2 (Table 2). In trial 1, the number of exuviae was significantly lower in the azadirachtin, buprofezin, and novaluron treatments than in the pyriproxyfen and nontreated control treatment for the 1^st^-2^nd^ instars (Fig 2A). For the 3^rd^-4^th^ instars, significantly fewer exuviae were observed in the azadirachtin treatment than in the pyriproxyfen and novaluron treatments, with the nontreated control showing the fewest (Fig 2A). In trial 2, the number of exuviae of 1^st^-2^nd^ instars was significantly lower in the buprofezin treatment than in the novaluron treatment, and was also lower than in the pyriproxyfen and nontreated control treatments (Fig 2B). For 3^rd^-4^th^ instars, the number of exuviae was significantly lower in the azadirachtin, buprofezin, and novaluron treatments than in the pyriproxyfen treatment, which in turn was lower than in the nontreated control (Fig 2B). For the 5^th^ instars, significantly fewer exuviae were observed in the buprofezin treatment than in the other treatments (Fig 2B).

#### Longevity.

**Fig 3:**
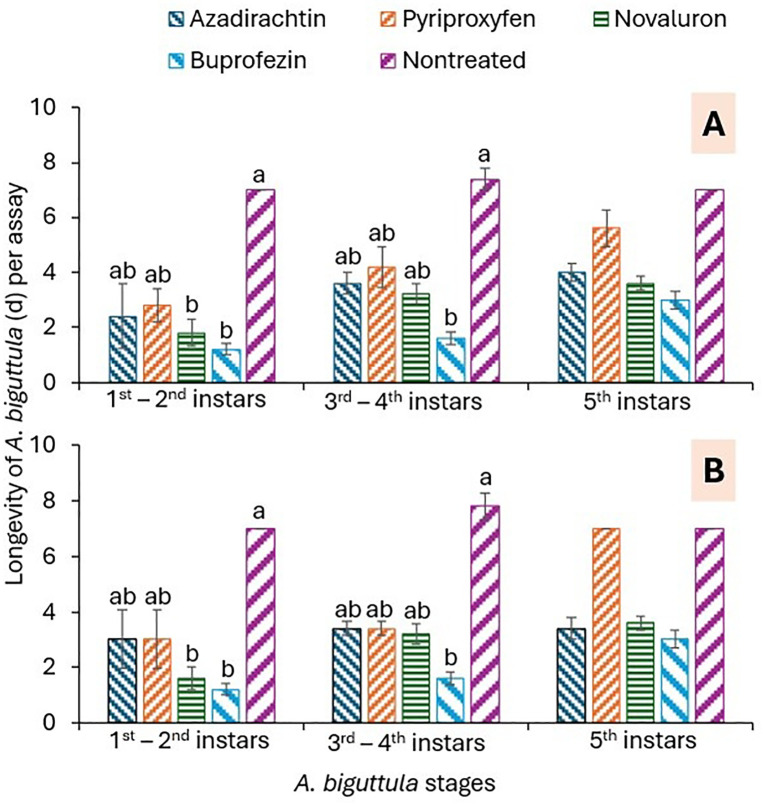
Mean (± SEM) number of days *A. biguttula* survived by stages in (A) trial 1 and (B) trial 2 in the leaf dip assay. Bars within each figure that share the same letters within each *A. biguttula* stage for the treatments are not significantly different at α = 0.05 (Tukey-Kramer test). Where no differences were observed among treatments, no letters are given.

In trial 1, the longevity of 1^st^-2^nd^ instars was significantly lower for the buprofezin and novaluron treatments than for the nontreated control treatment (*F* = 5.9; 4,16; *P* = 0.004; Fig 3A). For the 3^rd^-4^th^ instars, the number of days to survival was significantly lower in the buprofezin treatment than in the nontreated control (*F* = 4.3; 4,16; *P* = 0.015; Fig 3A). For 5^th^ instars, there was no significant difference between treatments in trial 1 (*F* = 2.3; df = 4, 16; *P* = 0.103; Fig 3A). In trial 2, the number of 1^st^-2^nd^ instars was significantly lower for the buprofezin and novaluron treatments than for the nontreated control treatment (*F* = 5.8; 4,16; *P* = 0.004; Fig 3B). The number of days to the 3rd-4th instar for survivors was significantly lower under buprofezin treatment than under the nontreated control (*F* = 5.1; 4,16; *P* = 0.007; Fig 3B). There was a significant difference between treatments (*F* = 3.4; 4,16; *P* = 0.034); however, there was no significant difference according to the Tukey-Kramer test (Fig 3B).

### IGR leaf smear assay with adjuvants on nymphal *A. biguttula* stage

#### Survival.

**Fig 4:**
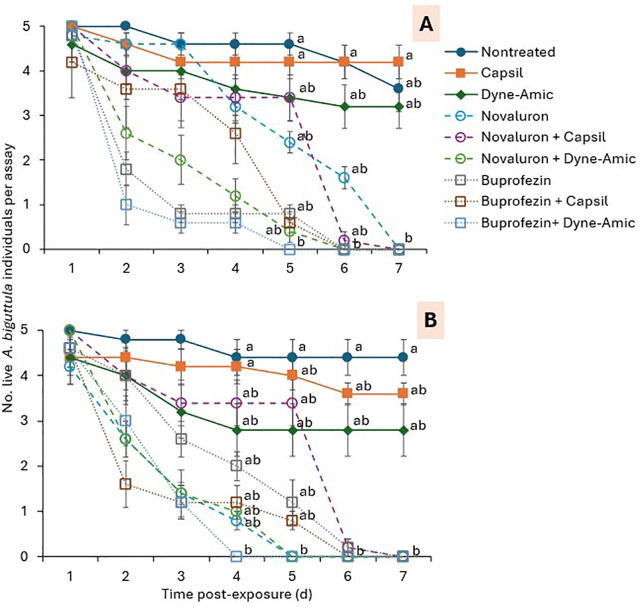
Mean (± SEM) number of 1^st^-2^nd^ *A. biguttula* survived in (A) trial 1 and (B) trial 2 in the leaf smear assay. Data points within each figure that share the same letters at the same post-exposure observation time for the treatments are not significantly different at α = 0.05 (Tukey-Kramer test). Where no differences were observed among treatments, no letters are given.

The effects of IGRs, exposure time, and their interaction on the survival of 1^st^-2^nd^ instars of *A. biguttula* differed significantly between trials 1 and 2 (Table 3). In trial 1, the number of 1^st^-2^nd^ instars was significantly lower in the buprofezin + dyne-amic treatment than in the nontreated control treatment at 5 DPE (Fig 4A). At 6 DPE, the number of 1^st^-2^nd^ instars was significantly lower in the buprofezin, buprofezin + dyne-Amic, buprofezin + Capsil, and novaluron + dyne-Amic treatments than in the nontreated control treatment. At the 7 SPE, all buprofezin and novaluron treatments, with and without adjuvants, had significantly fewer 1st-2nd instars than the nontreated control, the Capsil-only treatment, and the Dyne-Amic-only treatment (Fig 4A). In trial 2, the number of 1^st^-2^nd^ instars was significantly lower in the buprofezin + dyne-Amic treatment than in the nontreated control treatment at 4 DPE (Fig 4B). At 5 DPE, a significantly lower number of 1^st^-2^nd^ instars was observed in the buprofezin + dyne-Amic, novaluron, and novaluron + dyne-amic treatments than in the nontreated control treatment at 5 DPE (Fig 4B). At 6 and 7 DPE, the number of 1^st^-2^nd^ instars was significantly lower in all buprofezin and novaluron treatments, with or without adjuvant, than in the nontreated control (Fig 4B).

**Table 3:** Statistical analysis of *A. biguttula* survival and exuviae generated after exposure to various IGRs in a leaf smear assay.

Treatments	Trial 1			Trial 2		
	*F*	df	*P*	*F*	df	*P*
*1*^*st*^ *−2*^*nd*^ *instars*						
Insecticide	17.8	7,252	<0.001	18.9	8,284	<0.001
Time	20.9	7,252	<0.001	29.7	7,284	<0.001
Insecticide × Time	2.0	49,252	<0.001	1.9	56,284	<0.001
Exuviae						
Insecticide	6.6	8,284	<0.001	7.6	8,284	<0.001
Time	2.4	7,284	0.022	3.6	7,284	<0.001
Insecticide × Time	0.3	56,284	1.000	0.2	56,284	<0.001

#### Exuviae.

**Fig 5:**
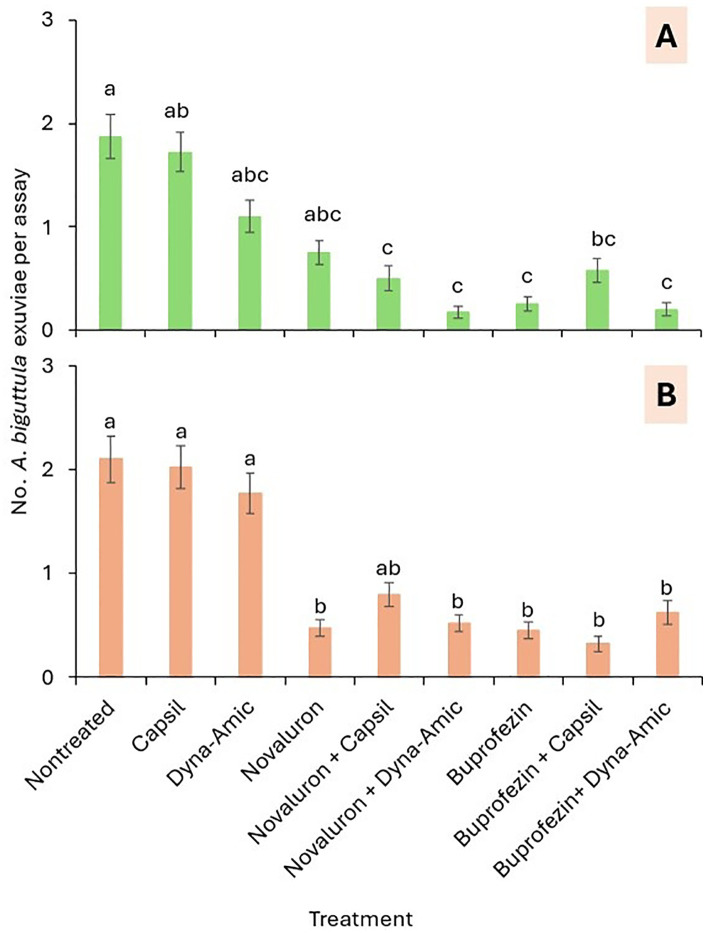
Mean (± SEM) number of *A. biguttula* exuviae in (A) trial 1 and (B) trial 2 in the leaf smear assay. Bars within each figure that share the same letters within each *A. biguttula* stage for the treatments are not significantly different at α = 0.05 (Tukey-Kramer test).

The effects of IGRs, exposure time, and their interaction on *A. biguttula* exuviae at the 1^st^-2^nd^ instar stages differed significantly between trials 1 and 2 (Table 3). In trial 1, the number of exuviae was significantly lower in the novaluron + Capsil, novaluron + Dyne-Amic, buprofezin, and buprofezin + Dyne-Amic treatments than in the nontreated control treatment (Fig 5A). In trial 2, the number of exuviae was significantly lower in all novaluron and buprofezin treatments, with and without adjuvant treatments, than in the nontreated control and the adjuvant-only treatments, except for novaluron + Capsil treatment (Fig 5B).

## Discussion

The present study provides the first comprehensive evaluation of the lethal effects of four IGRs: pyriproxyfen, novaluron, azadirachtin, and buprofezin on multiple developmental stages of *A. biguttula*. Among the tested compounds, buprofezin and novaluron consistently caused the highest mortality across developmental stages. The survival patterns observed in both trials of the leaf dip assay revealed clear differences among IGRs and developmental stages. In the earliest instars (1^st^-2^nd^), buprofezin and novaluron rapidly reduced survival within 24 h post-exposure, whereas pyriproxyfen and azadirachtin produced more moderate and delayed effects. These findings are consistent with previous studies showing that early instars of hemipterans are particularly vulnerable to chitin biosynthesis inhibitors due to their high molting frequency and thin cuticle structure [7, 18]. The strong early mortality induced by buprofezin is expected, as this compound disrupts chitin deposition during molting, leading to lethal molting failure. For 3^rd^-4^th^ instars, a similar pattern was observed: buprofezin and novaluron consistently produced the lowest survival across both trials, while pyriproxyfen and azadirachtin showed weaker effects. This stage-dependent sensitivity mirrors results reported for other hemipteran pests, such as *Bemisia tabaci* (Gennadius, 1889) (Hemiptera: Aleyrodidae) and *Nezara viridula* (Linnaeus, 1758) (Hemiptera: Pentatomidae), in which 2^nd^ and 3^rd^ instars exhibit intermediate susceptibility to IGRs [22]. In the 5^th^ instar and adult stages, mortality was generally lower and occurred more slowly, particularly with pyriproxyfen and azadirachtin. This reduced susceptibility is expected, as late instars molt less frequently and adults do not molt at all, limiting the physiological windows during which IGRs can exert their effects [21]. Nevertheless, novaluron and buprofezin still reduced survival in both trials, indicating that these compounds can disrupt cuticle formation and metabolic processes even in late developmental stages.

However, it is important to note that these observations are based on laboratory assays conducted at a single (maximum-label) rate and therefore do not permit quantitative comparisons of intrinsic toxicity among products or across developmental stages. The results should thus be interpreted as indicative of relative performance under the specific conditions tested, rather than definitive measures of comparative potency.

The exuviae data provide critical insight into the developmental disruption caused by IGRs. Across all instars and both trials, buprofezin and novaluron consistently produced the fewest exuviae, indicating strong inhibition of molting. This is consistent with their classification as chitin biosynthesis inhibitors, which prevent successful ecdysis and lead to arrested development [23,24]. The reduction in exuviae observed with azadirachtin, although less pronounced, is consistent with its known interference with ecdysteroid signaling and feeding behavior [25]. While reduced exuviae production suggests strong developmental disruption, this metric alone does not allow inference about long-term population-level outcomes such as reduced adult emergence or reproductive success, which were not measured in the present study.

The strong suppression of molting observed in the 1^st^ and 2^nd^ instars is particularly important, as early developmental disruption can have disproportionate impacts on population growth [26]. Reduced molting frequency in early instars has been shown to delay development, reduce adult emergence, and lower reproductive output in several hemipteran pests [7,18]. However, such population-level effects were not directly assessed here and should therefore be interpreted cautiously. The consistent reduction in exuviae across both trials suggests potential for delayed development under laboratory conditions, but does not, by itself, demonstrate population suppression in operational systems such as nurseries or field environments.

The longevity assays further support the disruptive effects of IGRs on *A. biguttula* development. In both trials, buprofezin and novaluron significantly reduced the number of days those early instars survived, reflecting the lethal molting failures observed in the survival and exuviae data. The reduced longevity of 3^rd^-4th instars under buprofezin treatment is consistent with previous findings in other leafhopper species, in which chitin synthesis inhibitors shorten lifespan by inducing incomplete molts or cuticular defects [27]. (Horowitz and Ishaaya, 2004). Interestingly, longevity effects in 5th in stars were less pronounced, reflecting the reduced molting frequency at this stage. However, the trend toward shorter survival under buprofezin and novaluron treatments suggests that these compounds may also interfere with physiological processes beyond molting, such as cuticle hardening or metabolic homeostasis. These findings, however, are limited to survival duration under controlled laboratory conditions and do not account for ecological factors that may influence longevity and fitness in field populations. Although distinguishing between instars is challenging, this experiment tracks the same individual insects over time, allowing simultaneous assessment of mortality and longevity, and providing valuable baseline information on developmental disruption by IGRs.

The leaf smear assay provided additional insight into the performance of IGRs under conditions mimicking residue exposure on the adaxial leaf surface. Although mortality was slightly greater in treatments combining IGRs with Dyne-Amic® or Capsil®, the overall improvement in efficacy was limited, indicating that adjuvants did not substantially enhance IGR performance under the conditions tested. These results should be interpreted as assay-specific outcomes and may not fully reflect field performance, where environmental variability and insect behavior can influence exposure.

Consistent with this interpretation, the reduction in exuviae observed in the smear assay, particularly in novaluron- and buprofezin-treated samples, was primarily attributable to the IGRs’ mode of action (IRAC Groups 15 and 16). Our results indicate that adjuvants did not enhance the transovarial effects of novaluron or buprofezin against *A. biguttula*. Nymphal densities on leaves treated with novaluron or buprofezin alone were comparable to those observed when these IGRs were applied in combination with adjuvants, indicating no added benefit from adjuvant inclusion. This pattern is consistent with previous findings by Joseph [28], who reported that adjuvants did not improve the transovarial activity of novaluron against *Scirtothrips pyrioides* (Karny, 1910) (Thysanoptera: Thripidae), as similar reductions in nymphal densities were obtained with novaluron applied alone or with adjuvants. However, transovarial effects were not directly quantified in this study and should therefore be interpreted with caution.

More broadly, the present study does not evaluate dose–response relationships, field efficacy, or non-target effects, which are essential components for assessing the role of IGRs in integrated pest management (IPM) programs and resistance management strategies [29]. In addition, recent phylogeographic evidence indicates that the U.S. population of *A. biguttula* originates from a single-haplotype incursion, suggesting low initial genetic diversity, which may have important implications for both susceptibility patterns and the development of resistance under selection pressure [14]. The choice of bioassay method is critical for accurately assessing the efficacy and operational suitability of IGRs. The leaf dip method, which involves immersing leaves in an insecticide solution and allowing them to air-dry before introducing test insects, provides uniform coverage and maximizes exposure via both contact and ingestion. This method is particularly effective for evaluating systemic and translaminar insecticides, as it simulates field application and ensures consistent exposure across replicates. The leaf smear method, in contrast, involves applying insecticide solution directly to the leaf surface, often resulting in less uniform coverage and reduced efficacy, especially for compounds with limited translaminar movement. In the present study, leaf dip assays consistently produced higher mortality and greater developmental disruption than leaf smear assays, underscoring the importance of application method in determining operational outcomes. However, both methods remain laboratory-based and do not fully replicate field exposure pathways, including environmental degradation, plant growth dynamics, and insect movement behavior. Accordingly, while the observed patterns provide useful baseline information on efficacy, they should not be interpreted as direct evidence of field-level population suppression or operational performance. Similarly, implications regarding reduced reliance on broad-spectrum insecticides, resistance management, or sustainability outcomes are beyond the scope of the present study and require validation through dose–response analyses, multi-rate testing, and field-based evaluations.

Collectively, the results of this study demonstrate that IGRs, particularly buprofezin and novaluron, exhibited activity under laboratory conditions at the tested rate, affecting survival, molting, and development across multiple instars. These findings provide baseline efficacy data that can inform future research, but should not be extrapolated to infer long-term population suppression or comparative intrinsic toxicity. Future studies should incorporate concentration gradients to estimate LC50/LC90 values, assess reproductive parameters, and evaluate field performance and non-target impacts to better define the role of these compounds in IPM programs. The stage-dependent effects observed here also highlight the importance of timing in IGR applications. Early instars were consistently the most susceptible, suggesting that monitoring and early intervention may be important considerations in practical settings, although this hypothesis requires validation in the field.

## Conclusion

This study provides baseline efficacy data, demonstrating that buprofezin and novaluron effectively disrupt survival and development across multiple instars in the laboratory. While these results suggest potential utility for IPM programs, statements regarding sustainable IPM integration, population suppression in nursery or field systems, and long-term resistance management are beyond the scope of this work and should be interpreted with caution. Future research should evaluate dose–response relationships, LC50/LC90 values, field performance, impacts on natural enemies, and the risk of resistance development to support evidence-based management strategies. These findings provide preliminary laboratory insights and a foundation for further studies, but do not establish definitive operational recommendations.

The modes of action of IGRs remain highly selective for insect developmental pathways, resulting in stage-specific effects observed in the laboratory; their potential compatibility with biological control agents and non-target safety requires validation under field conditions.
